# Comparative genomics identifies a *cyl*-like region in *Streptococcus sinensis* isolates from a patient with infective endocarditis

**DOI:** 10.3389/fmicb.2026.1990134

**Published:** 2026-10-19

**Authors:** Wei Ma, Yanjun Zhang, Xiaochun Huang, Yuxiang Wan, Changzi Deng, Jia Lin, Li Xu, Qin Qin

**Affiliations:** 1 Department of Laboratory Diagnostics, Changhai Hospital, Second Military Medical University, Shanghai, China; 2 Department of Disease Prevention and Control, Changhai Hospital, Second Military Medical University, Shanghai, China

**Keywords:** comparative genomics, *cyl*-like region, infective endocarditis, orthogroups, *Streptococcus sinensis*, virulence genes

## Abstract

*Streptococcus sinensis* is an uncommon member of the viridans group streptococci reported in infective endocarditis (IE), but its genomic characteristics remain incompletely defined. We performed whole-genome sequencing of three *S. sinensis* isolates recovered from blood culture and valvular tissue from a single patient with IE and compared them with 199 public *Streptococcus* genomes. Orthology analysis, phylogenetic reconstruction, SNP analysis, VFDB and CARD screening, gene-specific BLAST, Pfam/HMMER analysis, read mapping, synteny comparison, *in silico* structural modeling, and erythrocyte hemolysis assays were integrated. The three isolates differed by only 0–1 SNPs and formed a near-clonal group. Core single-copy ortholog phylogeny placed *S. sinensis* closest to *S. cristatus*, followed by *S. sanguinis* and *S. gordonii*. The three isolates retained the host-interaction-associated genes *eno*, *pavA*, and *plr*/*gapA* and carried an identical 11,341-bp, 12-gene *cyl*-like block comprising 11 *cyl*-like genes and *acpC*. Core-gene phylogeny analysis indicated that the distribution of the complete block was lineage-associated. Gene-specific BLAST and read mapping confirmed full-length sequence conservation and complete mapped-base coverage of the block, while Pfam/HMMER supported enzyme- and transporter-associated domain assignments for several encoded proteins. Structural modeling predicted soluble enzyme-like folds for CylG-like and CylZ-like proteins and a membrane-permease-like architecture with transmembrane helices for CylB-like. On Columbia sheep-blood agar, all three clinical isolates showed visible hemolytic zones and exhibited detectable hemolytic activity in a sheep erythrocyte hemolysis assay. All three *S. sinensis* isolates shared a type 1 CARD-derived resistance-marker profile containing only *Spne_parC_FLO*. These findings clarify the phylogenomic position of the near-clonal clinical isolates and identify a shared *cyl*-like genomic region accompanied by measurable hemolytic activity, providing a basis for further investigation of their host-interaction and pathogenic potential.

## 1 Introduction

Infective endocarditis (IE) is a severe cardiovascular infection. Despite advances in diagnosis, antimicrobial therapy, and surgery, IE continues to carry high in-hospital and long-term mortality (Ambrosioni et al., 2017; Feng et al., 2025; Holland et al., 2016; Murdoch et al., 2009). Viridans group streptococci are important causes of community-acquired IE, particularly in patients with underlying valvular disease. *Streptococcus sinensis* was first isolated and named in 2002 from the blood culture of a patient with IE in Hong Kong, China (Woo et al., 2002). Subsequent cases have been reported in several countries and regions (Tabone Adami et al., 2024; Pan et al., 2024; Wang et al., 2025). Although few cases have been documented, its recurrent association with IE suggests that *S. sinensis* may possess genomic features relevant to endocardial infection that remain incompletely understood.

The taxonomic position of *S. sinensis* has also been debated. Early analyses based on 16S rRNA and single genes did not consistently resolve its relationship to streptococci associated with the Mitis, Anginosus, or Sanguinis groups (Woo et al., 2002, 2006). Whole-genome analyses subsequently showed that *S. sinensis* forms a relatively distinct lineage within the genus *Streptococcus* and is closely related to *S. cristatus* and *S. oligofermentans* (Jensen et al., 2016; Teng et al., 2014). Core-genome phylogeny therefore provides a more stable framework for defining its taxonomic position.

Streptococci have large accessory gene pools, and gene gain, gene loss, and recombination contribute to ecological adaptation and the evolution of pathogenicity (Lefébure and Stanhope, 2007; Medini et al., 2005; Tettelin et al., 2005). For *S. sinensis*, the central question extends beyond taxonomic placement to whether the organism carries genetic features that merit further investigation in the setting of IE. We addressed this question through five complementary analyses: orthogroup composition, core-genome phylogeny, single-nucleotide polymorphism (SNP) variation, virulence and antimicrobial resistance gene profiles, and synteny of the *cyl*-like genomic region.

## 2 Materials and methods

### 2.1 Clinical isolates, genome sequencing, and bioinformatic workflow

Three clinical isolates of *S. sinensis* (418A, 418B, and 433) were recovered from the same patient with IE. Isolates 418A and 418B were recovered from the same blood culture, whereas isolate 433 was recovered from valvular vegetation/necrotic tissue. Species identification was performed by matrix-assisted laser desorption/ionization time-of-flight mass spectrometry (MALDI-TOF MS; Microflex LT, Bruker Daltonics, Germany). The clinical laboratory report contained disk-diffusion zone diameters for seven agents and a penicillin G MIC determined by broth microdilution. Antimicrobial susceptibility results of the strains were interpreted according to the Clinical and Laboratory Standards Institute M100, 32nd edition.

Genomic DNA was extracted from each isolate and subjected to paired-end sequencing on an Illumina NovaSeq 6000 platform. Sequencing depth exceeded 100× for each sample. Orthologous gene analysis was performed with OrthoFinder v2.4.0, and SNP analysis was performed with Snippy. Core single-copy gene sequences were aligned with MAFFT and trimmed with Gblocks. A maximum-likelihood phylogeny was inferred with FastTree and annotated and visualized with iTOL. Antimicrobial resistance and virulence genes were predicted against CARD and VFDB, respectively, and heatmaps and summary plots were generated in R. Linear comparisons of the *cyl*-like region and its 41,404-bp flanking genomic window were generated with Easyfig v2.2.3.

### 2.2 Reference genome collection and orthology analysis

We downloaded 199 *Streptococcus* reference genomes selected from BV-BRC for multispecies comparison (Olson et al., 2023). The metadata contained 20 named *Streptococcus* species and one unassigned *Streptococcus* sp. NPS 308 genome; together with the three clinical isolates, these genomes formed a 202-genome dataset. Public genomes were preferentially selected when BV-BRC classified them as Good quality and as either whole-genome shotgun or complete genome assemblies. For the CARD resistance-marker analysis, the matrix included 198 genomes (195 public reference genomes and the three clinical isolates); four rat-derived public genomes were excluded from that analysis. Recorded metadata included species, strain, host, isolation source, sequencing platform, assembly status, contig count, genome size, GC content, CheckM completeness and contamination, and accession number. Metadata for the 199 public *Streptococcus* reference genomes are provided in Supplementary Table 3.

OrthoFinder v2.4.0 was used to identify orthologs and cluster gene families (Emms and Kelly, 2019), which were then categorized as core, accessory, or genome-specific. Orthogroup functions were classified with the COG database (Galperin et al., 2021). Orthogroup composition, pan-genome accumulation curves, and COG classifications were visualized in R. Because the dataset comprised multiple *Streptococcus* species, these analyses describe gene-pool characteristics within a multispecies comparative framework and were not used to infer whether the *S. sinensis* intraspecies pan-genome is open.

### 2.3 SNP and phylogenetic analyses

SNP analysis of single-copy orthologs was performed with Snippy. Pairwise SNP counts were subsequently calculated from the SNP alignment with snp-dists v0.8.2, and the SNP matrix and clustering heatmap were generated in R. For phylogenetic analysis, core single-copy gene sequences identified by the orthology analysis were aligned with MAFFT (Katoh and Standley, 2013), trimmed with Gblocks (Castresana, 2000), and used to infer a maximum-likelihood tree with FastTree (Price et al., 2010). Species, group, and source metadata were annotated and visualized with iTOL (Letunic and Bork, 2021). The original 202-tip tree is shown in Supplementary Figure 11. For a readable main-text view, that same Newick tree was pruned to one representative public genome per non-sinensis taxon, selected as the within-taxon tree-distance medoid, while all seven *S. sinensis* genomes were retained. No new phylogeny was inferred; the induced topology and pairwise patristic distances among retained tips were preserved. Because the original core-SNP dataset contained too few sites to provide a stable representation of evolutionary relationships, the core single-copy gene tree was used as the principal phylogenetic result.

### 2.4 Virulence genes, antimicrobial resistance genes, and *cyl*-like region analysis

Antimicrobial resistance and virulence-associated homologues were predicted against CARD and VFDB, respectively (Alcock et al., 2020; Chen et al., 2005), and the resulting matrices were visualized in R. Throughout this study, “not detected” denotes no qualifying homologous match under the applied database and threshold settings; it does not establish absolute gene absence or deletion of an alternative adhesion or virulence mechanism. This distinction is particularly important for a sparsely characterized organism such as *S. sinensis*, because database representation, sequence divergence, threshold selection, and fragmented assemblies can all affect detection. The routine VFDB screen used an 80% similarity threshold. Presence/absence calls for adhesion-associated genes and *cyl*-like genes are provided in Supplementary Table 2, and the full screening matrix is provided in Supplementary Table 4. Because some members of the *cyl*-like locus did not meet the routine VFDB threshold, 11 prespecified *cyl*-like calls in Supplementary Figure 8 and Supplementary Table 2 were supplemented using locus-specific annotation corroborated by BLAST and synteny analyses; these targeted calls are not equivalent to threshold-qualified VFDB hits.

For gene-specific verification, the 12 coding sequences of the SSSC01 locus in GCF_030586725.1 (*cylX-cylD-cylG-acpC-cylZ-cylA-cylB-cylE-cylF-cylI-cylJ-cylK*) were used as queries in BLAST+ v2.15.0 with an *E*-value threshold of 1 × 10^–5^ and otherwise default settings. The corresponding block in each clinical assembly spans Scaffold10 coordinates 20,397–31,737 (11,341 bp). Linear comparisons of this block and the broader flanking genomic window were generated with Easyfig v2.2.3 (Sullivan et al., 2011). The earlier 9,635-bp comparison subinterval from 418A Scaffold10 (coordinates 21,547–31,181; *cylG* through *cylJ*) and the 41,404-bp flanking window (coordinates 8,353–49,756) were retained as operational Easyfig windows. Because the 12 target coding sequences showed 100% nucleotide identity and 100% query and subject coverage between GCF_030586725.1 and each clinical assembly, the corresponding public SSSC01 protein sequences were used to represent the identical clinical sequences in a profile-HMM domain scan. Proteins were analyzed with the EMBL-EBI HMMER web API using hmmscan against Pfam 37.2 (24,076 profile models; accessed 20 September 2026) (Potter et al., 2018; Mistry et al., 2021). A profile was accepted only when both the full-sequence score and the best-domain score met the model-specific Pfam gathering thresholds. Full-sequence and domain *E*-values, bit scores, query coverage, model coverage, and threshold values are reported in Supplementary Table 5. These Pfam models evaluate broad protein-family or domain architecture and are not dedicated gene-specific *cyl* models; failure to pass a Pfam threshold was not interpreted as gene absence or loss of function. To evaluate read support for the locus, quality-controlled reads from each isolate were aligned to the corresponding assembly with BWA-MEM v0.7.17 using default parameters, and depth was calculated with SAMtools. Scaffold10 was 84,521 bp in each assembly. Gene-wise mean depth and mapped-base coverage were summarized for the 12 target coding sequences (Supplementary Table 5).

For *in silico* structural visualization, representative CylG-like (240 aa), CylZ-like (157 aa), and CylB-like (292 aa) protein sequences encoded in the 9,635-bp region were modeled. The loci were sequence-identical in 418A, 418B, and 433; therefore, one representative sequence was modeled for each protein. Structures were predicted with the ESMFold web API (accessed July 2026; Lin et al., 2023). Mean pLDDT was calculated from the PDB B-factor field across Cα atoms. Putative transmembrane segments displayed in Supplementary Figure 9 were inferred with a Kyte-Doolittle hydropathy scan (19-residue window, threshold 1.6, minimum segment length 12 residues). These models were used only to visualize annotation plausibility and were not interpreted as evidence of protein expression, enzymatic activity, transport, hemolysis, or virulence.

### 2.5 Colony observations and hemolysis assay

For the plate photographs, cultures were grown on Columbia sheep-blood agar for 48 h at 35 °C in 5% CO_2_. For the liquid assay, isolates were activated on Columbia blood agar and grown in Todd–Hewitt broth (THB). Bacterial suspensions were prepared in PBS and adjusted to OD_600_ = 0.4. Washed sheep erythrocytes were resuspended to 1% (v/v), giving a final erythrocyte concentration of 0.5% after equal-volume mixing. Equal volumes (500 μL each) of bacterial suspension and erythrocyte suspension were mixed and incubated at 37 °C for 60 min. After centrifugation, absorbance of the supernatant was measured at 541 nm. The negative control contained PBS and erythrocytes; the positive control used a 0.1% SDS solution before equal-volume mixing (0.05% final SDS concentration). A clinical *S. agalactiae* strain was included as a comparator. Three parallel tubes of the three isolates were measured in one experiment. Relative signal was calculated from group means as 100 × (mean sample OD_541_ − mean PBS OD_541_)/(mean SDS-control OD_541_ − mean PBS OD_541_). Raw technical-tube OD_541_ measurements and the exploratory PBS-corrected, SDS-normalized group mean signals are provided in Supplementary Table 6.

## 3 Results

### 3.1 Clinical course

A 26-years-old man with no history of immunodeficiency, diabetes, or other underlying diseases was admitted to our hospital. Two weeks prior to admission, he had developed intermittent pain in the right groin and right foot, which was poorly relieved by rest, accompanied by night sweats, weight loss (5 kg over 2 weeks), and fever. He had previously visited the spinal surgery department, where lumbar MRI revealed mild bulging of the L4/5 intervertebral disk and mild posterior protrusion of the L5/S1 intervertebral disk. Treatment with cobamamide, diosmin, and folic acid had been administered with limited efficacy. Notably, the patient had experienced dental pain with fever several weeks earlier, which had improved after oral metronidazole therapy.

On admission, the patient was febrile (39 °C), tachycardic (heart rate 120 beats/min), and tachypneic (respiratory rate 26 breaths/min), with a cachectic appearance. Laboratory investigations revealed leukocytosis (white blood cell count 14.20 × 10^9^/L), elevated inflammatory markers (erythrocyte sedimentation rate 49 mm/h; C-reactive protein 68.63 mg/L), anemia (hemoglobin 88 g/L), hypoalbuminemia (albumin 28 g/L), and mildly elevated procalcitonin (0.204 ng/mL). Transthoracic echocardiography demonstrated mitral valve vegetations. All four bottles from a set of blood cultures yielded *S. sinensis*. A small pericardial effusion was identified on the 4th day of admission. Given the patient’s history of penicillin allergy, vancomycin was initiated for antimicrobial therapy. On the 7th day of admission, the patient underwent mechanical mitral valve replacement. Intraoperatively, large vegetations were observed on both the anterior and posterior mitral leaflets, with perforation of the anterior mitral leaflet. *S. sinensis* was again recovered from intraoperative valvular necrotic tissue.

Postoperatively, the patient continued to exhibit transient fluctuations in inflammatory markers and fever, including a temperature of 39 °C on the 19th day of admission (Figure 1). The archived susceptibility records for the blood-culture and tissue isolates reported susceptible categories for vancomycin, linezolid, chloramphenicol, penicillin G, clindamycin, ceftriaxone, erythromycin, and levofloxacin; the original zone diameters and the available penicillin G MICs are provided in Supplementary Table 1. The vancomycin regimen was adjusted in stages: 500 mg every 6 h on 30 November, every 12 h from 1 to 7 December, and every 8 h from 8 to 28 December. The trough concentration measured on 23 December was 8.97 μg/mL. Postoperative anticoagulation and intravenous levofloxacin were administered for 1 month, followed by supportive care. The patient was discharged after recovery following a 28-days hospitalization.

**FIGURE 1:**
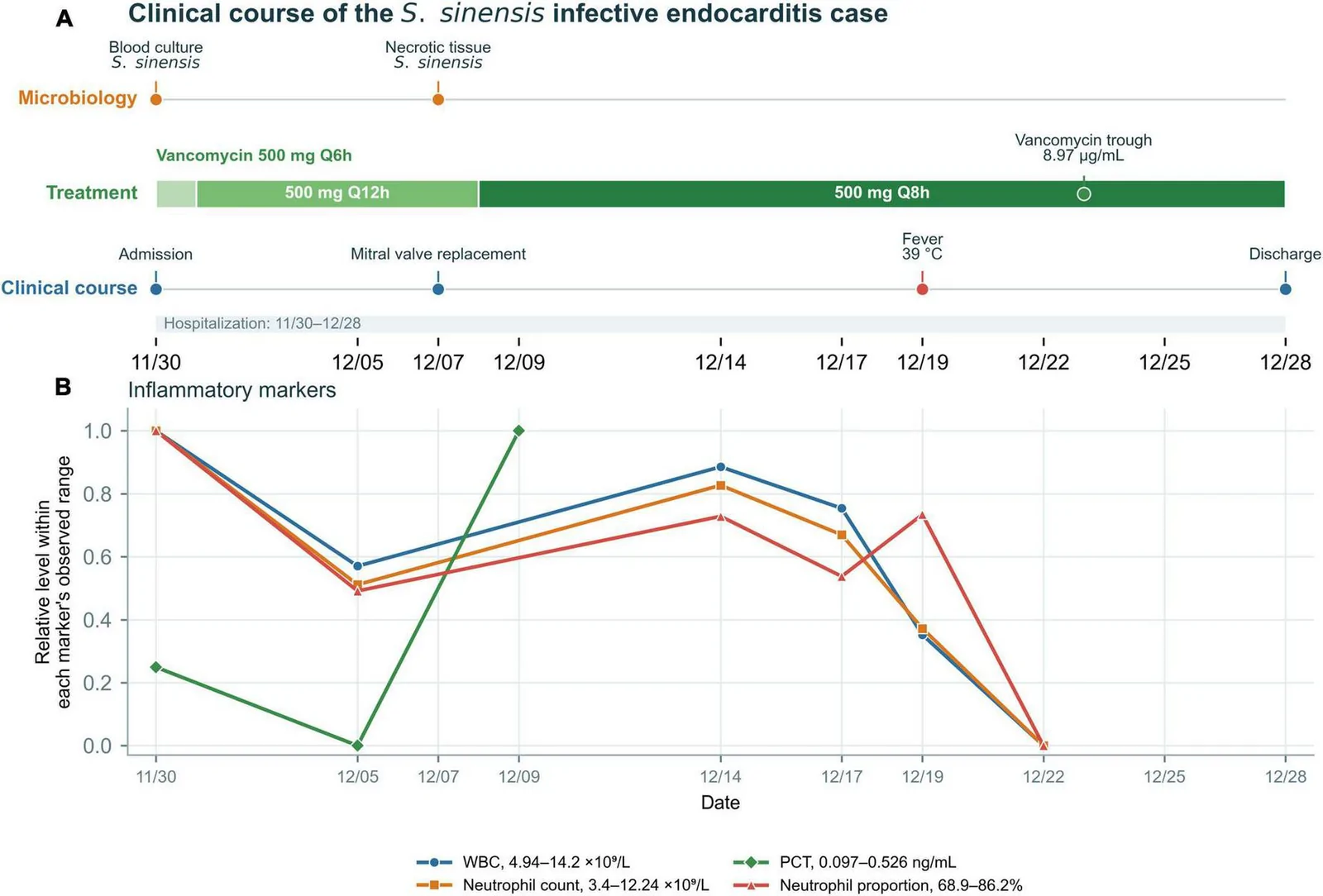
Clinical timeline and inflammatory-marker dynamics in the patient with infective endocarditis. **(A)** Blood cultures, culture of valvular necrotic tissue, surgery, fever, and staged vancomycin treatment. **(B)** Longitudinal changes in white blood cell count, procalcitonin, neutrophil count, and neutrophil percentage. Each biomarker was independently min–max normalized over its observed values: 0 represents its measured minimum and 1 its measured maximum, not a zero concentration or a common physical unit. Only observed time points are shown; no measurement was carried forward after its last available time point.

### 3.2 A small core and a large accessory gene pool characterize the multispecies *Streptococcus* dataset

OrthoFinder identified 7,200 orthogroups across the 202 *Streptococcus* genomes. Of these, 382 occurred in all genomes, 79 were detected in only one genome, and the remaining 6,739 occurred in more than one but fewer than all 202 genomes. The small core and predominant accessory component indicate that the species represented in this dataset retain extensive variable gene content beyond a conserved genomic backbone. This distribution is consistent with the dynamic genome structure reported for *Streptococcus*.

The pan-genome accumulation analyses continued to increase as additional genomes were included and did not reach a stable plateau. Thus, the 20 named *Streptococcus* species and one unassigned *Streptococcus* sp. NPS 308 sampled here did not encompass all gene families detectable within this dataset. Chao1 estimates, accumulation and rarefaction analyses, and COG functional classifications supported the same overall interpretation (Supplementary Figures 1–5).

### 3.3 The core single-copy gene phylogeny resolves the position of *S. sinensis*

The original 202-tip core single-copy ortholog phylogeny resolved the principal *Streptococcus* lineages in the dataset (Supplementary Figure 11). In a representative 27-tip view pruned from the same tree (Figure 2), *S. sinensis* occupied a lineage associated with the Mitis group and was closest to *S. cristatus*; neighboring lineages included oral streptococci such as *S. sanguinis* and *S. gordonii*. The three clinical isolates clustered with public *S. sinensis* reference genomes and did not fall outside the species lineage. This topology agrees with previous whole-genome studies describing *S. sinensis* as a distinct phylogenetic lineage (Jensen et al., 2016; Teng et al., 2014).

**FIGURE 2:**
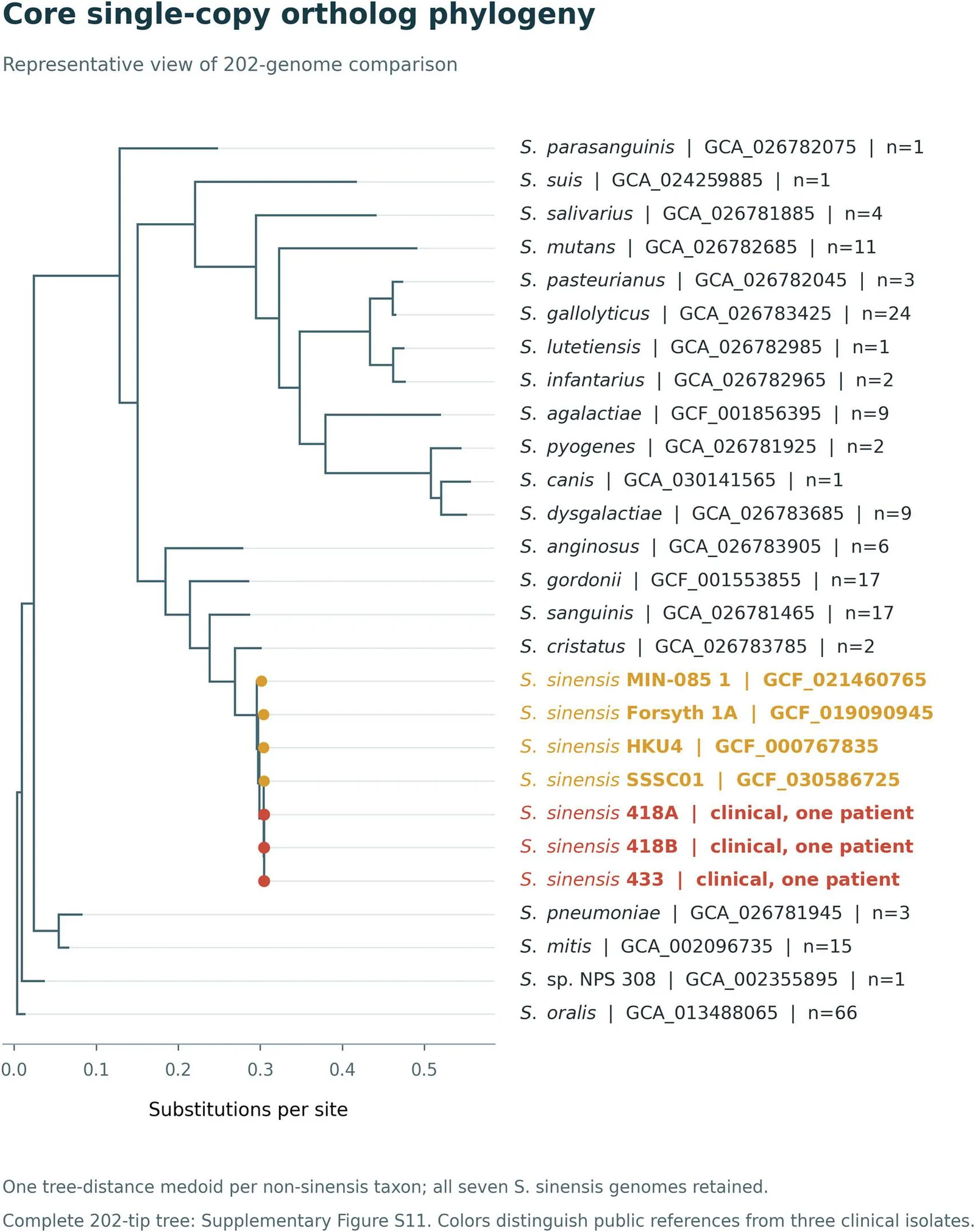
Representative view of the core single-copy ortholog phylogeny. A representative 27-tip view was generated by pruning the original 202-tip Newick tree while retaining all seven *S. sinensis* genomes and one public genome for each remaining taxon, selected as the within-taxon tree-distance medoid. No new phylogeny was inferred; the induced topology, branch lengths, and pairwise patristic distances among the retained tips were preserved. Numbers in parentheses indicate the number of public genomes assigned to each non–*S. sinensis* taxon in the original dataset. The complete 202-tip phylogeny is provided in Supplementary Figure 11.

### 3.4 SNP variation supports within-species clustering and between-species differentiation

The SNP clustering heatmap based on single-copy orthologs showed a grouping pattern consistent with the core-gene phylogeny (Supplementary Figure 7). Isolates from the same species generally formed neighboring clusters, whereas SNP differences increased across species. *S. sinensis* occupied a region adjacent to closely related streptococci, including *S. cristatus*, supporting their proximity at both the core-genome and SNP levels. The interspecies SNP boxplot further showed that within-species variation was lower than between-species variation (Supplementary Figure 6).

Pairwise SNP distances confirmed that the three clinical isolates represented repeated sampling of one near-clonal infection. Isolates 418A and 418B differed by 0 SNPs, and isolate 433 differed from each by 1 SNP (Figure 3). The three isolates therefore do not constitute independent epidemiological observations.

**FIGURE 3:**
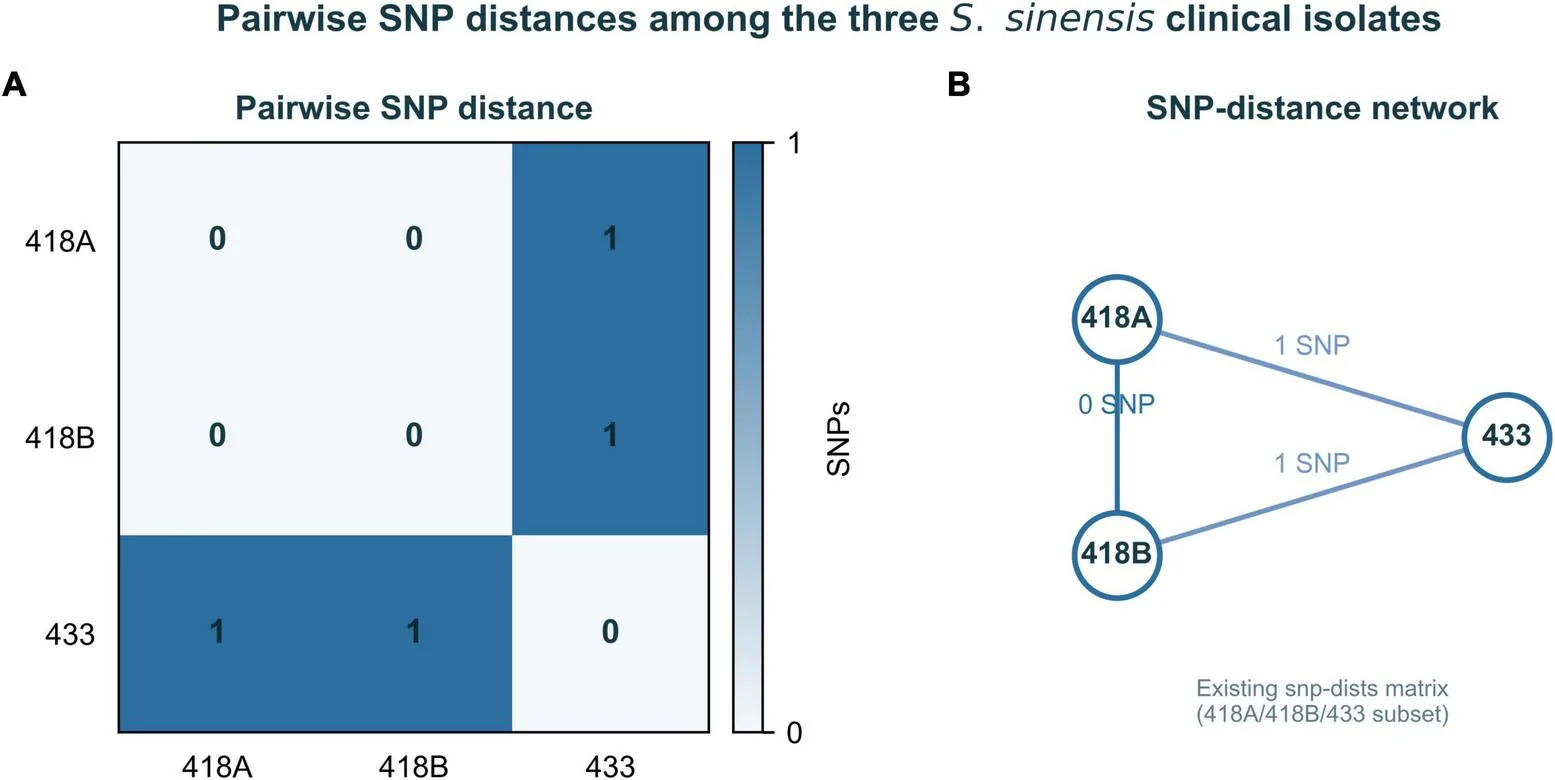
Pairwise SNP distances among the three clinical *S. sinensis* isolates. **(A)** Heatmap extracted from the snp-dists distance matrix. **(B)** SNP-distance network. Isolates 418A and 418B differed by 0 SNPs, whereas isolate 433 differed from each by 1 SNP.

### 3.5 Virulence-associated homologue screening and the *cyl*-like region in *S. sinensis*

The VFDB matrix, supplemented with targeted calls for the *cyl*-like locus, showed that virulence-associated homologue profiles varied with species background (Supplementary Figure 8). Under the applied threshold, *S. sinensis* genomes yielded fewer qualifying VFDB hits overall, and no detected homologue was unique to *S. sinensis*. In all three clinical isolates, no qualifying matches were obtained for the Gtf system (*gtfB*, *gtfC*, *gtfD*, or *gtfG*) or for canonical adhesins including *cshA*/*cshB*, *spaP*/*pac*, *gbpA*/*gbpD*, *gspB*/*hsa*, and *pblA*/*pblB*. In contrast, *eno*, *pavA*, and *plr*/*gapA* were detected in all three isolates (Supplementary Table 2). These threshold-dependent results describe database matches only and do not demonstrate that *S. sinensis* lacks adhesion or other virulence mechanisms.

All three clinical isolates carried the same targeted cyl-like block. Routine VFDB screening identified cylB, cylG, and cylZ homologues associated with the beta-hemolysin/cytolysin system of *S. agalactiae* (Supplementary Figure 8 and Supplementary Table 2). Gene-specific BLAST additionally verified cylA, cylD, cylE, cylF, cylI, cylJ, cylK, and cylX, together with acpC, in all three clinical assemblies. For every one of the 12 coding sequences, query coverage, subject coverage, and nucleotide identity relative to SSSC01 (GCF_030586725.1) were each 100%. Independent read mapping supported these calls: all 12 target coding sequences had 100% mapped-base coverage, with gene-wise mean depths of 568.6–678.4× in 418A, 549.0–702.4× in 418B, and 210.3–250.6× in 433 (Supplementary Table 5). These targeted calls are reported separately from threshold-qualified VFDB hits and do not establish gene expression or function. Pfam/HMMER supported broad enzyme- or transporter-associated domain classes in CylG-like, AcpC-like, CylZ-like, CylA-like, CylB-like, CylF-like, CylI-like, and CylJ-like proteins. No available Pfam profile passed both curated gathering thresholds for CylX-like, CylD-like, CylE-like, or CylK-like; this result indicates the absence of a qualifying Pfam domain assignment, not the absence of the BLAST-verified coding sequence. Across the seven-genome *S. sinensis* comparison, the complete targeted profile occurred in the three near-clonal clinical isolates and GCF_030586725.1 but was not detected in HKU4 (GCF_000767835.1), Forsyth 1A (GCF_019090945.2), or MIN-085 (GCF_021460765.1), demonstrating that the block is not universal within the species.

To describe this intraspecific distribution in phylogenetic context, we mapped the detection status of the three threshold-qualified markers *cylB*, *cylG*, and *cylZ* onto a local single-copy core-gene phylogeny of the seven available *S. sinensis* genomes (Figure 4). The three near-clonal clinical isolates and GCF_030586725.1 simultaneously harbored these three markers and formed a closely related clade. HKU4 (GCF_000767835.1), Forsyth 1A (GCF_019090945.2), and MIN-085 (GCF_021460765.1) had no qualifying matches for all three markers.

**FIGURE 4:**
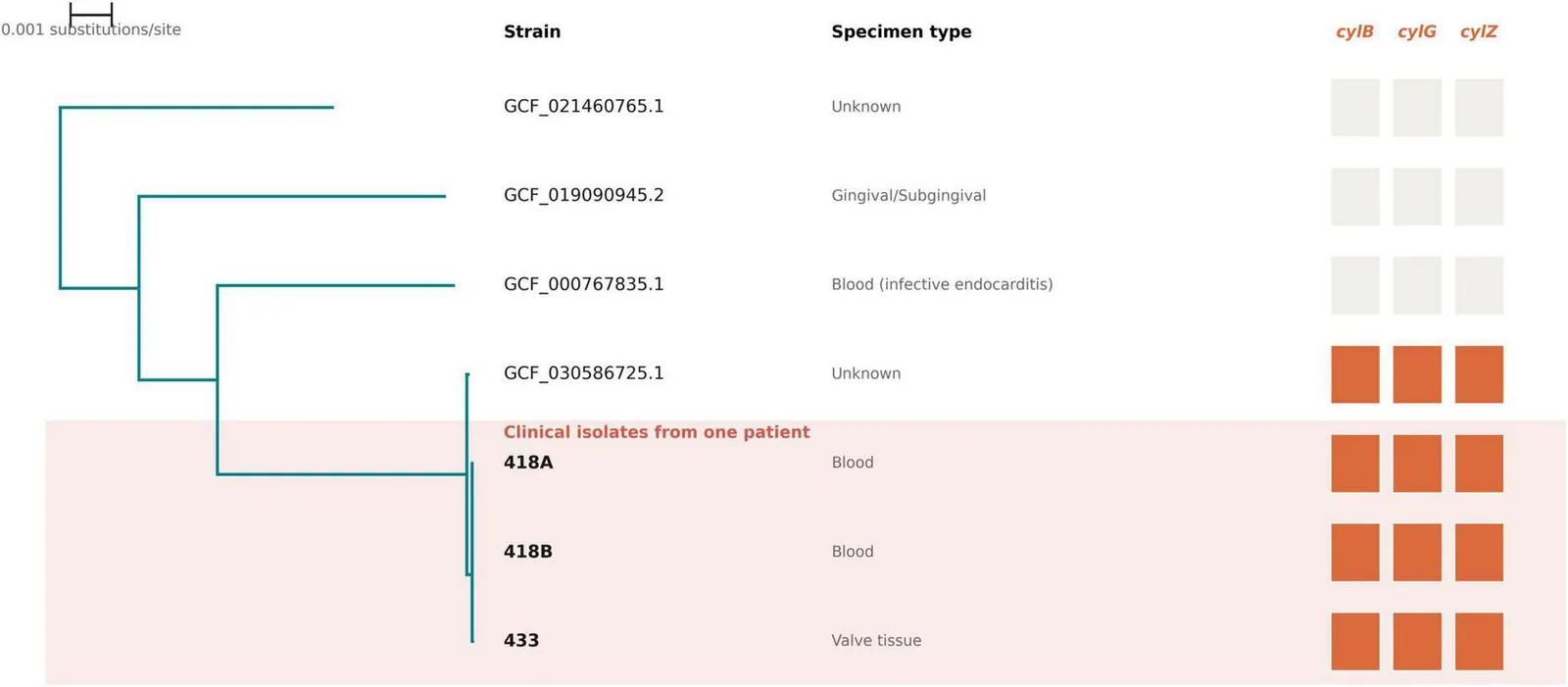
Core-gene phylogeny and distribution of threshold-qualified *cyl*-like markers among the seven included *S. sinensis* genomes. The phylogeny is annotated by specimen type and VFDB matches for *cylB*, *cylG*, and *cylZ*. Orange cells indicate matches meeting the applied screening thresholds, whereas pale cells indicate no qualifying match and do not demonstrate gene absence. The three near-clonal clinical isolates and GCF_030586725.1 formed a clade in which all four genomes yielded qualifying matches for the three markers. No qualifying matches were obtained for HKU4 (GCF_000767835.1), Forsyth 1A (GCF_019090945.2), or MIN-085 (GCF_021460765.1). These database-screening results do not establish gene expression, function, or an association with infective endocarditis.

### 3.6 The *cyl*-like region shares a conserved local genomic context across *Streptococcus* species

Because the three isolates differed by only 0-1 SNPs, isolate 418A was used as the representative in the synteny figure. Gene-specific BLAST confirmed the complete 12-gene block (cylX-cylD-cylG-acpC-cylZ-cylA-cylB-cylE-cylF-cylI-cylJ-cylK) at Scaffold10 coordinates 20,397–31,737 in 418A, 418B, and 433. For every gene in each isolate, query coverage, subject coverage, and nucleotide identity relative to the SSSC01 reference were 100%. The complete block therefore spans 11,341 bp, whereas the previously defined 9,635-bp interval (21,547–31,181) is an internal comparison subinterval rather than the full targeted block. Scaffold10 was 84,521 bp in all three assemblies; the block began 20,396 bp from the left end and ended 52,784 bp before the right end, so it was not adjacent to a scaffold boundary. When the query was expanded to the 41,404-bp flanking genomic window, all three isolates retained complete matches.

Cross-species linear comparisons showed that *S. sinensis* shared a broadly similar *cyl*-like locus organization with *S. gallolyticus*, whereas *S. gordonii* retained only a partial corresponding region (Figure 5A). Comparison of the operational 41,404-bp flanking genomic window among selected *S. sinensis* genomes showed that HKU4 and Forsyth 1A did not contain the same complete interval (Figure 5B). Comparison of the broader flanking context further showed conservation of some flanking genes with *S. cristatus* and a different genomic context for the canonical *S. agalactiae cyl* cluster (Figure 5C).

**FIGURE 5:**
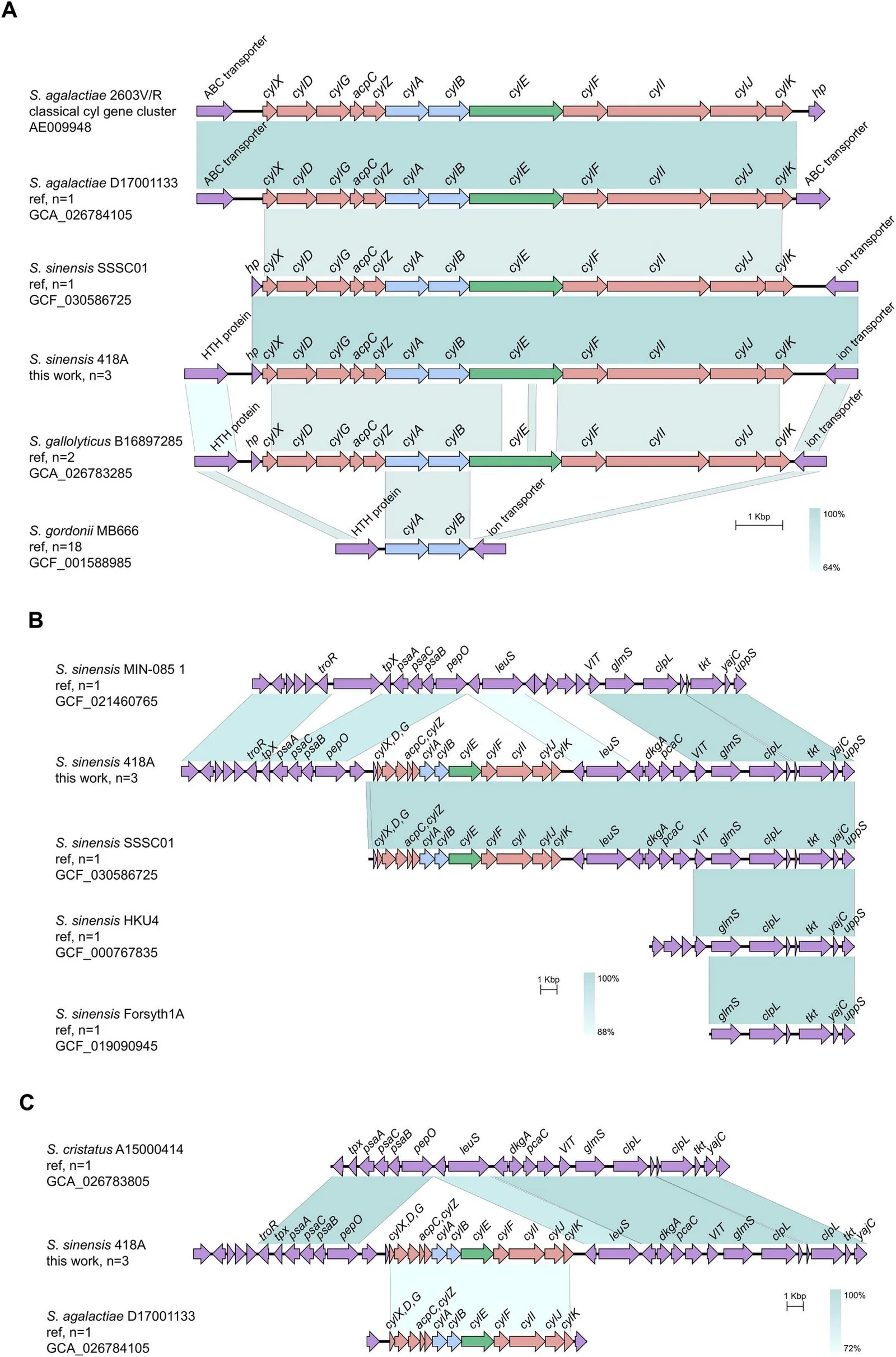
Comparative organization of the *S. sinensis cyl*-like block and its flanking genomic context. Isolate 418A is displayed as the representative of the three near-clonal isolates from one patient. **(A)** Comparison of the complete targeted 12-gene block (*cylX-cylD-cylG-acpC-cylZ-cylA-cylB-cylE-cylF-cylI-cylJ-cylK*; 11,341 bp in the clinical assemblies) with corresponding regions from *S. agalactiae*, *S. gallolyticus*, and *S. gordonii*. **(B)** Comparison of the operational 41,404-bp flanking genomic window among selected *S. sinensis* genomes; HKU4 and Forsyth 1A did not contain the same complete interval. **(C)** Comparison of the broader flanking context with *S. cristatus* and *S. agalactiae*. Shaded links indicate homologous sequence blocks. The 41,404-bp interval is an operational comparison window.

### 3.7 *In silico* structural visualization of selected *cyl*-like proteins

*In silico* structural visualization of the three selected *cyl*-like proteins is provided as Supplementary Figure 9. The CylG-like and CylZ-like proteins were predicted to adopt soluble enzyme-like folds, whereas the CylB-like protein contained predicted transmembrane helices compatible with a membrane-permease architecture. The mean pLDDT scores were 98.3 for CylG-like, 84.3 for CylZ-like, and 95.6 for CylB-like. These computational models provide structural context for sequence annotations only; they do not establish protein expression, enzymatic activity, transport, hemolysis, cellular injury, or virulence in *S. sinensis*.

### 3.8 CARD screening shows a limited predicted resistance-marker profile in the clinical isolates

CARD analysis identified 41 antimicrobial resistance genes or mutation markers among the 198 genomes included in the resistance matrix after exclusion of four rat-derived assemblies. *Spne_parC_FLO* was most frequent and occurred in 163 genomes; *patA*, *patB*, *pmrA*, RlmA(II), Smit_CdsA_DAP, and penicillin-binding protein-associated markers were also common. Predicted resistance-marker burdens differed among species, with *S. oralis*, *S. pneumoniae*, and *S. mitis* carrying more markers than most other streptococci (Supplementary Figure 10). These results represent genomic predictions and do not substitute for phenotypic antimicrobial susceptibility testing (Alcock et al., 2020; Guérin et al., 2000).

The predicted *S. sinensis* resistance profile was relatively restricted. All three clinical isolates were assigned to type 1, in which *Spne_parC_FLO* was the only detected marker. Type 1 also predominated among public *S. sinensis* genomes, whereas a small number of reference strains additionally carried *mel* and were assigned to type 28. Unlike *S. pneumoniae*, *S. mitis*, and *S. oralis*, *S. sinensis* did not show enrichment of multidrug-resistance gene combinations.

### 3.9 Colony and hemolysis assays

*Streptococcus sinensis* strains 418A and 418B were recovered from blood, whereas isolate 433 was recovered from valvular vegetation/necrotic tissue. On Columbia sheep-blood agar, the plate photograph showed larger colonies assigned to 418A and smaller colonies assigned to 418B on the same plate; all three isolates produced hemolytic zones, with *S. agalactiae* included as a comparator. In the liquid assay, mean supernatant OD_541_ values (mean ± SD of three technical tubes) were 0.083 ± 0.025 for PBS, 1.267 ± 0.105 for the SDS control, 0.500 ± 0.145 for the *S. agalactiae* comparator, 0.397 ± 0.021 for 418A, 0.613 ± 0.229 for 418B, and 0.327 ± 0.136 for 433. After correcting group means to the PBS reference and scaling to the SDS reference, the relative signals were 35.2%, 26.5%, 44.8%, and 20.6%, respectively (Figure 6).

**FIGURE 6:**
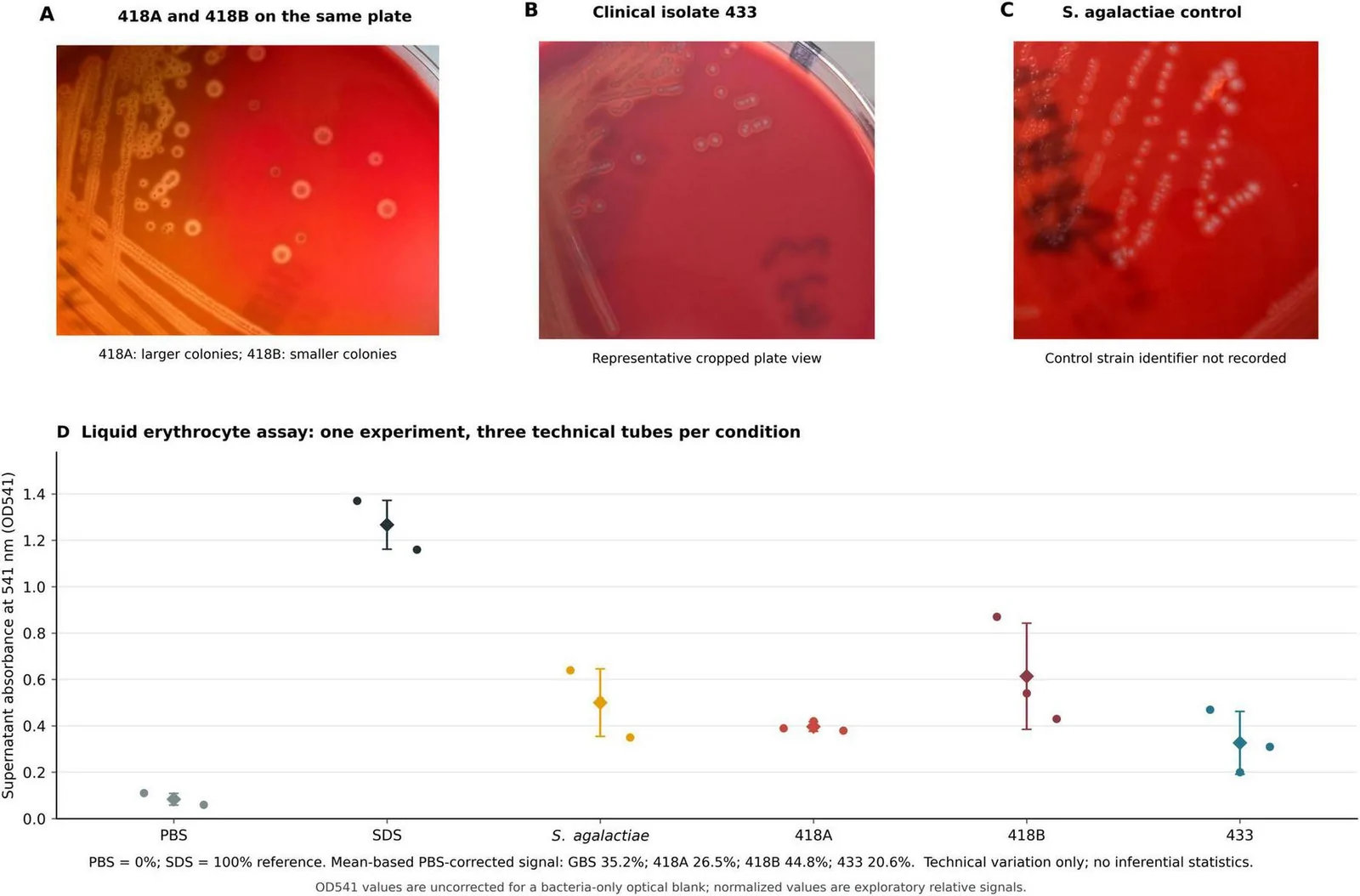
Colony and erythrocyte hemolysis assays observations. **(A)** Cropped view of the shared plate showing the larger-colony morphotype assigned to 418A and the smaller-colony morphotype assigned to 418B. **(B)** Clinical isolate 433. **(C)** *S. agalactiae* comparator. Plate images are representative crops photographed after growth on Columbia sheep-blood agar for 48 h at 35 °C in 5% CO2; they are not calibrated for quantitative comparison of colony or halo diameter. Clear β-hemolytic zones were visually apparent. **(D)** Supernatant OD_541_ after mixing 500 μL bacterial suspension with 500 μL of 1% washed sheep erythrocytes and incubating for 60 min at 37 °C. Dots show three technical tubes from one experiment; diamonds and error bars show mean ± SD. PBS and SDS are negative and positive references, respectively; SDS was 0.1% before mixing and 0.05% in the final reaction. Relative signals are calculated from group means as 100 × (sample – PBS)/(SDS – PBS). No bacteria-only optical blank was available. These exploratory measurements do not establish a *cyl*-dependent mechanism.

## 4 Discussion

Infective endocarditis remains a life-threatening cardiac infection. The Global Burden of Disease 2021 analysis estimated 77,844 IE-related deaths worldwide in 2021 (Miao et al., 2025). Contemporary studies continue to report in-hospital mortality of approximately 15%–20% (Brien et al., 2025), rising above 60% when IE is complicated by shock (Pirianu-Masgras et al., 2025). Viridans group streptococci remain important causes of IE, particularly in community-acquired disease and in patients with underlying valvular abnormalities (Holland et al., 2016; Murdoch et al., 2009).

*Streptococcus sinensis* is a rare member of the viridans group streptococci and has been reported in Asia, Europe, and North America (Tabone Adami et al., 2024; Pan et al., 2024; Wang et al., 2025). Nearly all available clinical isolates have been recovered from patients with IE. A 2025 literature review summarized 12 cases in patients aged 8–63 years (median, approximately 40 years); many underwent valve replacement or related surgery because of severe underlying mitral regurgitation (Wang et al., 2025). The patient in the present study likewise underwent mechanical mitral valve replacement and received a temporary intraoperative pacemaker.

Two lines of evidence support an oral origin for *S. sinensis*. Clinically, some reported patients with *S. sinensis* IE had concurrent odontogenic disease (Pan et al., 2024; Wang et al., 2025; Zhang et al., 2022). Microbiologically, the organism has been detected by multiplex PCR in subgingival plaque from patients with severe localized chronic periodontitis (de Lillo et al., 2006). *S. sinensis*-associated 16S rRNA signals have also been reported in gastric mucosal and fecal microbiome studies, although viable isolates have not been recovered from these sites (Aviles-Jimenez et al., 2014; Toscano et al., 2017). The scarcity of reported human infections outside IE raises the possibility that *S. sinensis* carries factors favoring colonization of or damage to cardiac valves.

Most reported patients had favorable outcomes after β-lactam-based therapy, including ampicillin, amoxicillin, or penicillin G, often combined with gentamicin (Pan et al., 2024; Wang et al., 2025). Available susceptibility data indicate that *S. sinensis* is susceptible to most agents tested, particularly β-lactams. In this case, a documented history of penicillin allergy led to staged vancomycin dosing: 500 mg every 6 h on 30 November, every 12 h from 1 to 7 December, and every 8 h from 8 to 28 December, together with surgical management. The 2023 ESC endocarditis guideline identifies vancomycin as an alternative when beta-lactams cannot be used in patients with documented allergy, while noting that beta-lactams are preferred when feasible (Delgado et al., 2023). The observed pre-dose vancomycin concentration of 8.97 μg/mL on 23 December was below the 10–15 mg/L pre-dose range listed in that guideline. However, only one concentration was available, and no AUC-based monitoring, vancomycin MIC, body weight, or contemporaneous renal-function data were available. Pharmacokinetic adequacy therefore cannot be determined retrospectively from this value, and the favorable clinical outcome cannot be attributed to vancomycin exposure alone. CARD analysis indicated a low predicted resistance burden, with *Spne_parC_FLO* as the sole marker in the three isolates. Because all three isolates remained phenotypically susceptible to levofloxacin, this marker should not be interpreted as evidence of phenotypic fluoroquinolone resistance in *S. sinensis*. In related viridans streptococci, a single *parC* alteration can be associated with lower-level fluoroquinolone resistance, whereas higher-level resistance typically requires alterations in more than one quinolone target (Guérin et al., 2000). This relationship has not been established in *S. sinensis*; no *gyrA* mutations were detected in the three isolates.

The pathogenic basis and phylogenetic position of *S. sinensis* remain incompletely resolved. Comparisons between MALDI-TOF MS and *sodA* sequencing in other *Streptococcus* species complexes have shown that a single identification method may not fully resolve closely related taxa (Hinse et al., 2011). Early 16S rRNA analyses placed *S. sinensis* near *S. gordonii* (96.4% identity) and *S. intermedius* (96.3% identity) (Woo et al., 2002), and one hypothesis proposed that *S. sinensis* represents a common ancestor of the Anginosus and Mitis groups (Woo et al., 2006). Because single genes or limited marker sets provide unstable bacterial classifications, whole-genome analyses offer a stronger basis for taxonomic placement. Phylogenomic and MALDI-TOF MS analyses led to the proposal of a “sinensis group” containing *S. sinensis*, *S. oligofermentans*, and *S. cristatus* (Teng et al., 2014), while subsequent work identified *S. oligofermentans* as a later heterotypic synonym of *S. cristatus* (Jensen et al., 2016). Whether the proposed sinensis group clusters more closely with the Sanguinis or Mitis group remains uncertain. In our 20-species comparative framework, *S. sinensis* occupied a Mitis group-associated lineage and was closest to *S. cristatus*, followed by *S. sanguinis* and *S. gordonii*.

The limited number of core orthogroups and the predominance of accessory orthogroups illustrate extensive gene-content variation among *Streptococcus* species (Lefébure and Stanhope, 2007; Medini et al., 2005; Tettelin et al., 2005). This background makes gene acquisition, gene loss, and local genome remodeling plausible contributors to variation in *S. sinensis*. For oral streptococci associated with IE, persistence on damaged valves depends partly on interactions with host proteins, extracellular matrix, and platelet-fibrin vegetations. Oral streptococci use polysaccharide synthases, antigen I/II family proteins, serine-rich repeat glycoproteins, and surface or moonlighting proteins during biofilm formation, platelet interactions, and tissue colonization (Nobbs et al., 2009). In *S. gordonii*, the serine-rich repeat glycoproteins GspB and Hsa bind sialylated glycans on platelet glycoprotein Ibα and provide a well-characterized mechanism of platelet interaction relevant to IE (Bensing et al., 2004). Under the VFDB settings used here, *gspB*/*hsa*, *spaP*/*pac*, *gbpA*/*gbpD*, and *gtfB*/*gtfC*/*gtfD*/*gtfG* did not yield qualifying matches in the clinical isolates. The lack of qualifying matches for these canonical factors does not demonstrate that *S. sinensis* lacks adhesion or invasive capacity. All three clinical isolates carried homologues of *eno*, *pavA*, and *plr*/*gapA*. In other streptococci, surface α-enolase binds plasminogen (Pancholi and Fischetti, 1998), and pneumococcal PavA binds fibronectin and contributes to virulence (Holmes et al., 2001). The corresponding *S. sinensis* proteins are candidate host-interaction factors, but their expression and phenotypic roles in this species remain untested. More broadly, the present screen is constrained by the completeness of VFDB for under-characterized taxa, sequence divergence from curated reference factors, the selected similarity threshold, and assembly fragmentation; it cannot provide an exhaustive inventory of *S. sinensis* virulence mechanisms.

The *cyl*-like region was conserved across the near-clonal clinical isolates, but its function remains uncertain. The three isolates carried the same 11,341-bp, 12-gene block within the 41,404-bp operational flanking window. Gene-specific BLAST demonstrated full-length, sequence-identical matches to the SSSC01 block, while Pfam/HMMER supported broad domain assignments for several predicted biosynthetic and transporter components. These findings support sequence conservation and annotation plausibility. The local context resembled corresponding regions in *S. gallolyticus* and *S. gordonii* but differed from the canonical *S. agalactiae cyl* cluster. In group B *Streptococcus*, *cyl* genes participate in β-hemolytic/cytolytic activity, pigment biosynthesis, and tissue-barrier injury (Armistead et al., 2020; Gottschalk et al., 2006; Rajagopal, 2009; Spellerberg et al., 1999; Whidbey et al., 2013). Equivalent functions have not been demonstrated in *S. sinensis*.

The ESMFold models were used solely as *in silico* structural visualizations of the sequence annotations. The predicted folds of CylG-like and CylZ-like and the predicted transmembrane segments of CylB-like are compatible with annotations inferred by comparison with the canonical *cyl* system of *S. agalactiae*. Hemolysis assays in solid and liquid media indicated that the three isolates exhibited hemolytic activity. The assays lacked a *cyl*-like-negative strain as a control; therefore, they cannot establish reproducible erythrocyte lysis or link the phenotype to the *cyl*-like genomic region. Moreover, these data do not establish enzymatic activity, transporter function, protein expression, granadaene production, cellular injury, or virulence in *S. sinensis*. Therefore, it should be emphasized that these are only computational predictions.

The local phylogeny indicates that the *cyl*-like marker set is not universally detected across the available *S. sinensis* genomes but is concentrated within a closely related clade. In particular, HKU4 and Forsyth 1A lacked the same marker set and comparison interval, despite belonging to the same species. This pattern may reflect ancestral inheritance, lineage-specific retention, or unresolved gain-and-loss events; it does not establish a relationship between the locus and IE and directly limits any generalization to *S. sinensis* as a species. Importantly, 418A, 418B, and 433 were recovered from one patient and differ by only 0–1 SNPs, so they cannot be regarded as three independent epidemiological observations. Future studies should compare independent, clinically characterized *S. sinensis* isolates with phylogenetically matched locus-negative controls and evaluate the region with transcriptional, mutagenesis, hemolytic/cytotoxicity, and infection-model experiments.

The current plate photographs showed conspicuous β-hemolytic zones, and the three isolates showed hemolytic activity in a preliminary sheep erythrocyte hemolysis assay, yielding OD_541_ signals between the PBS and SDS controls. This experiment contained three technical tubes and lacked a bacteria-only optical blank and a *cyl*-like-negative strain control, so it could not establish reproducible erythrocyte lysis or link the phenotype to the *cyl*-like block. The assay conditions also limit comparison with previous work. In *S. sinensis* Forsyth1A, CSP exposure did not significantly alter erythrocyte lysis in an assay using defibrinated rabbit erythrocytes (Brennan et al., 2022). A study of *S. agalactiae* cell-derived hemolysin measured sheep-erythrocyte lysis after 10 min at 37 °C and read released hemoglobin at OD_540_ (Gottschalk et al., 2006). The present assay used whole-cell suspensions, sheep erythrocytes, a 60-min incubation, and OD_541_. Differences in strain, assay material, erythrocyte source, and duration prevent direct comparison of the numerical signals. The higher mean signal for 418B than 418A is descriptive and should be interpreted cautiously given the single experiment and technical-only replication.

Published studies of Forsyth1A place these findings in the broader context of competence regulation. Although Forsyth1A lacks the complete *cyl*-like block detected in the clinical isolates, CSP activated *comX*, competence-associated transcription, and genetic transformation, demonstrating a functional ComABCDE competence-signaling circuit (Brennan et al., 2022). CSP exposure did not significantly alter crystal-violet biofilm formation or erythrocyte lysis under the tested conditions. Structure-activity analysis subsequently identified the N-terminal Asp1 residue as essential for *S. sinensis* CSP-ComD activation and showed that a D1A analogue acts as a competitive inhibitor (Renshaw et al., 2026). These studies establish competence signaling in *S. sinensis* but do not link ComABCDE to IE, the *cyl*-like block, or its regulation. Therefore, more research is required to clarify the pathogenic mechanisms of *S. sinensis.*

This study has five limitations. First, the three clinical isolates were recovered from one patient and were near-clonal; they cannot represent the population structure of *S. sinensis* or provide independent epidemiological evidence for an IE-associated locus. The absence of the *cyl*-like region from HKU4, Forsyth 1A, and other reference genomes further precludes a species-wide or IE-specific inference. Second, the present phenotypic evidence is preliminary: the sheep erythrocyte hemolysis assay was performed once with three technical tubes, and no bacteria-only optical blank or *cyl*-like-negative strain control was included. These data cannot establish reproducible hemolytic activity or link the phenotype to the *cyl*-like region; transcriptional analysis, targeted genetic tests, cytotoxicity assays, and infection models were not performed. Third, the 41,404-bp flanking window was defined on a short-read scaffold, with unresolved flanking structure; genomic-island boundaries, insertion site, atypical GC content, mobility, and related features were not established. Fourth, the routine VFDB screen was supplemented by gene-specific BLAST and Pfam/HMMER, but the available Pfam models provide broad domain-level rather than gene-specific functional evidence and did not yield threshold-passing assignments for every component. BWA-MEM read mapping showed 100% mapped-base coverage and high mean depth for every target coding sequence, and the block was located 20,396 bp and 52,784 bp from the two Scaffold10 ends. These mapping data support recovery of the sequence in the assemblies but do not establish transcription, protein expression, or biological function. Fifth, public genomes were heterogeneous in sample source, sequencing platform, and assembly quality, and the available clinical phenotypes, susceptibility results, and outcomes did not constitute a dataset suitable for statistical analysis. Additional independent isolates and functional studies are required before any pathogenic mechanism can be defined.

## 5 Conclusion

This comparative genomic analysis describes three near-clonal *S. sinensis* isolates recovered from one patient with infective endocarditis. The isolates carried a conserved 11,341-bp, 12-gene *cyl*-like block within an operationally defined 41,404-bp flanking window. Gene-specific BLAST and Pfam/HMMER strengthened the sequence-level and domain-level annotation of this locus but did not establish protein expression or function. The exploratory erythrocyte hemolysis assay yielded OD_541_ signals between the PBS and SDS controls, but one experiment with technical replicates cannot demonstrate a reproducible hemolytic phenotype or a causal role for the locus. The data identify a candidate *cyl*-like genomic region for further study associated with infective endocarditis. Independent strain collections, complete genome resolution, and functional experiments are required to define its biological significance.

## Data Availability

The datasets presented in this study can be found in online repositories. The names of the repository/repositories and accession number(s) can be found in the article/Supplementary material.

## Supplementary material

Supplementary Figure 1 Rarefaction curve of the Chao1 estimator for the multi-species *Streptococcus* genome dataset. Points show the mean Chao1 estimate at each sampling depth, and error bars show variation across resampling replicates.

Supplementary Figure 2 Pan-genome accumulation curves for the multi-species *Streptococcus* genome dataset based on 1,000 random permutations. Blue, green, and red lines represent the total pan-genome, core gene set, and genome-specific gene set, respectively; error bars show variation across permutations. The dashed line represents the fitted pan-genome curve.

Supplementary Figure 3 Pan-genome rarefaction curves of gene families at progressively stricter occurrence thresholds. Curves show the cumulative gene-family counts across sampling depths for all families and for families detected in at least 2, 3, 4, 5, or 6 genomes. Error bars show variation across resampling replicates.

Supplementary Figure 4 Numbers of COG-assigned gene families in the accessory, core, and genome-specific gene groups. Bars show counts by COG functional category; numeric labels indicate gene-family counts.

Supplementary Figure 5 Proportional COG functional-category composition of the accessory, core, and genome-specific gene groups. Each bar represents the percentage distribution of COG-assigned gene families within one gene group.

Supplementary Figure 6 Distribution of pairwise SNP differences within and between *Streptococcus* species based on the single-copy ortholog SNP matrix. Pairwise SNP counts were calculated with snp-dists v0.8.2. Boxes show the median and interquartile range, whiskers denote 1.5 times the interquartile range, and points represent pairwise comparisons. Colors identify species or comparison groups as indicated in the figure.

Supplementary Figure 7 Full clustering heatmap based on SNP differences in single-copy orthologs. Isolates from the same species generally clustered more tightly, whereas between-species differences were larger. This expanded matrix is provided as a supplementary figure because individual sample labels are not legible at standard main-text width.

Supplementary Figure 8 Full distribution matrix of virulence-associated gene calls in the 202-genome *Streptococcus* dataset. Cells denote gene detection status, and annotation bars summarize gene category and species. Values were generated by routine VFDB screening, except for 11 prespecified *cyl*-like genes supplemented using locus-specific annotation corroborated by BLAST and synteny analyses. Targeted calls are not equivalent to threshold-qualified VFDB hits, and a non-hit does not establish gene absence.

Supplementary Figure 9 *In silico* structural visualization of selected *cyl*-like proteins in the three clinical *S. sinensis* isolates. **(A)** Schematic organization of the 9,635-bp *cyl*-like region. **(B)** Predicted protein architectures of CylG-like, CylZ-like, and CylB-like proteins; black ticks indicate hydropathy-predicted transmembrane segments. **(C)** ESMFold-predicted three-dimensional models with mean pLDDT scores. One representative sequence was modeled for each protein because the corresponding clinical loci were sequence-identical. These computational predictions provide structural context for annotation only and do not constitute functional validation.

Supplementary Figure 10 Distribution of antimicrobial resistance genes and mutation markers predicted against CARD among 198 genomes after exclusion of four rat-derived assemblies. *S. sinensis* had a limited predicted resistance profile; all three clinical isolates were assigned to type 1, containing only *Spne_parC_FLO*. These are sequence-based predictions and do not substitute for phenotypic susceptibility testing.

Supplementary Figure 11 Complete 202-tip core single-copy ortholog phylogeny of the *Streptococcus* comparative dataset. The tree includes 199 public reference genomes and the three near-clonal clinical *S. sinensis* isolates. Rings annotate available taxonomic and sampling metadata. The original Newick tree was pruned to generate the representative main-text view in Figure 2; that view retains one tree-distance medoid per non-sinensis taxon and all seven *S. sinensis* genomes while preserving their induced topology and pairwise patristic distances. Individual tip labels in this complete view require magnification and should not be interpreted from standard page-width display.

Supplementary Table 1 Antimicrobial susceptibility results for *Streptococcus sinensis* isolates recovered from blood culture and valvular necrotic tissue.

Supplementary Table 2 Detection calls for adhesion-associated and *cyl*-like genes in the three clinical *S. sinensis* isolates. Values of 1 and 0 denote a qualifying match and no qualifying match, respectively, under the stated evidence source. Targeted *cyl*-like calls based on locus-specific annotation corroborated by BLAST and synteny are distinguished from routine threshold-qualified VFDB hits; a value of 0 does not establish gene absence.

Supplementary Table 3 Metadata for the 199 public *Streptococcus* reference genomes included in the multispecies comparative genomic analyses.

Supplementary Table 4 Virulence-associated homologue detection matrix for the 202-genome *Streptococcus* dataset used to generate Supplementary Figure 8. Values of 1 and 0 denote detected and not detected under the specified screening approach. Eleven prespecified *cyl*-like calls were supplemented using locus-specific annotation corroborated by BLAST and synteny; these targeted calls are not equivalent to routine threshold-qualified VFDB hits, and a value of 0 does not establish gene absence.

Supplementary Table 5 Targeted BLAST, read-mapping, and Pfam/HMMER verification of the *cyl*-like block.

Supplementary Table 6 Raw OD541 readings and exploratory PBS-corrected, SDS-normalized signals.
