# Supplementary material for: Comparative genomics identifies a cyl-like region in Streptococcus sinensis isolates from a patient with infective endocarditis
Source: Front Microbiol. 2026 Oct 19;17:1990134. doi: 10.3389/fmicb.2026.1990134 (PMC13724373; doi:10.3389/fmicb.2026.1990134)
Supplement: Supplementary file 1 [file Supplementary_file_1.docx]

The following are the captions of Supplementary Tables [2-4] :
Supplementary Table 2. Detection calls for adhesion-associated and *cyl*-like genes in the three clinical *S. sinensis* isolates. Values of 1 and 0 denote a qualifying match and no qualifying match, respectively, under the stated evidence source. Targeted *cyl*-like calls based on locus-specific annotation corroborated by BLAST and synteny are distinguished from routine threshold-qualified VFDB hits; a value of 0 does not establish gene absence.

Supplementary Table 3. Metadata for the 199 public *Streptococcus* reference genomes included in the multispecies comparative genomic analyses.

Supplementary Table 4. Virulence-associated homologue detection matrix for the 202-genome *Streptococcus* dataset used to generate Supplementary Figure 8. Values of 1 and 0 denote detected and not detected under the specified screening approach. Eleven prespecified *cyl*-like calls were supplemented using locus-specific annotation corroborated by BLAST and synteny; these targeted calls are not equivalent to routine threshold-qualified VFDB hits, and a value of 0 does not establish gene absence.
