# Supplementary material for: Comparative genomics identifies a cyl-like region in Streptococcus sinensis isolates from a patient with infective endocarditis
Source: Front Microbiol. 2026 Oct 19;17:1990134. doi: 10.3389/fmicb.2026.1990134 (PMC13724373; doi:10.3389/fmicb.2026.1990134)
Supplement: Supplementary file 2 [file Supplementary_file_2.docx]

**Supplementary Figure Legends**

Supplementary Figure S1. Rarefaction curve of the Chao1 estimator for the multi-species *Streptococcus* genome dataset. Points show the mean Chao1 estimate at each sampling depth, and error bars show variation across resampling replicates.

Supplementary Figure S2. Pan-genome accumulation curves for the multi-species *Streptococcus* genome dataset based on 1,000 random permutations. Blue, green, and red lines represent the total pan-genome, core gene set, and genome-specific gene set, respectively; error bars show variation across permutations. The dashed line represents the fitted pan-genome curve.

Supplementary Figure S3. Pan-genome rarefaction curves of gene families at progressively stricter occurrence thresholds. Curves show the cumulative gene-family counts across sampling depths for all families and for families detected in at least 2, 3, 4, 5, or 6 genomes. Error bars show variation across resampling replicates.

Supplementary Figure S4. Numbers of COG-assigned gene families in the accessory, core, and genome-specific gene groups. Bars show counts by COG functional category; numeric labels indicate gene-family counts.

Supplementary Figure S5. Proportional COG functional-category composition of the accessory, core, and genome-specific gene groups. Each bar represents the percentage distribution of COG-assigned gene families within one gene group.

Supplementary Figure S6. Distribution of pairwise SNP differences within and between *Streptococcus* species based on the single-copy ortholog SNP matrix. Pairwise SNP counts were calculated with snp-dists v0.8.2. Boxes show the median and interquartile range, whiskers denote 1.5 times the interquartile range, and points represent pairwise comparisons. Colors identify species or comparison groups as indicated in the figure.

Supplementary Figure S7. Full clustering heatmap based on SNP differences in single-copy orthologs. Isolates from the same species generally clustered more tightly, whereas between-species differences were larger. This expanded matrix is provided as a supplementary figure because individual sample labels are not legible at standard main-text width.

Supplementary Figure S8. Full distribution matrix of virulence-associated gene calls in the 202-genome *Streptococcus* dataset. Cells denote gene detection status, and annotation bars summarize gene category and species. Values were generated by routine VFDB screening, except for 11 prespecified *cyl*-like genes supplemented using locus-specific annotation corroborated by BLAST and synteny analyses. Targeted calls are not equivalent to threshold-qualified VFDB hits, and a non-hit does not establish gene absence.

Supplementary Figure S9. In silico structural visualization of selected *cyl*-like proteins in the three clinical *S. sinensis* isolates. (A) Schematic organization of the 9,635-bp *cyl*-like region. (B) Predicted protein architectures of CylG-like, CylZ-like, and CylB-like proteins; black ticks indicate hydropathy-predicted transmembrane segments. (C) ESMFold-predicted three-dimensional models with mean pLDDT scores. One representative sequence was modeled for each protein because the corresponding clinical loci were sequence-identical. These computational predictions provide structural context for annotation only and do not constitute functional validation.

Supplementary Figure S10. Distribution of antimicrobial resistance genes and mutation markers predicted against CARD among 198 genomes after exclusion of four rat-derived assemblies. *S. sinensis* had a limited predicted resistance profile; all three clinical isolates were assigned to type 1, containing only *Spne_parC_FLO*. These are sequence-based predictions and do not substitute for phenotypic susceptibility testing.

Supplementary Figure S11. Complete 202-tip core single-copy ortholog phylogeny of the *Streptococcus* comparative dataset. The tree includes 199 public reference genomes and the three near-clonal clinical *S. sinensis* isolates. Rings annotate available taxonomic and sampling metadata. The original Newick tree was pruned to generate the representative main-text view in Figure 2; that view retains one tree-distance medoid per non-sinensis taxon and all seven *S. sinensis* genomes while preserving their induced topology and pairwise patristic distances. Individual tip labels in this complete view require magnification and should not be interpreted from standard page-width display.
