# Supplementary material for: Comparative genomics identifies a cyl-like region in Streptococcus sinensis isolates from a patient with infective endocarditis
Source: Front Microbiol. 2026 Oct 19;17:1990134. doi: 10.3389/fmicb.2026.1990134 (PMC13724373; doi:10.3389/fmicb.2026.1990134)
Supplement: Supplementary file 3 [file Table_1.docx]

**Supplementary Table S1**

**Antimicrobial susceptibility results for *Streptococcus sinensis* isolates recovered from blood culture and valvular necrotic tissue**

**Blood culture isolate(s)**

| **Antimicrobial agent** | **Method** | **Recorded result** | **Recorded interpretation** | **CLSI M100 (32nd ed.) breakpoint range** |
| --- | --- | --- | --- | --- |
| Vancomycin | Disk diffusion | 26 mm | S | S ≥17 mm |
| Linezolid | Disk diffusion | 35 mm | S | S ≥21 mm |
| Chloramphenicol | Disk diffusion | 31 mm | S | S ≥21 mm; I 18–20 mm; R ≤17 mm |
| Penicillin G | Broth microdilution | 0.064 μg/mL | S | S ≤0.12 μg/mL; I 0.25–2 μg/mL; R ≥4 μg/mL |
| Clindamycin | Disk diffusion | 34 mm | S | S ≥19 mm; I 16–18 mm; R ≤15 mm |
| Ceftriaxone | Disk diffusion | 37 mm | S | S ≥27 mm; I 25–26 mm; R ≤24 mm |
| Erythromycin | Disk diffusion | 36 mm | S | S ≥21 mm; I 16–20 mm; R ≤15 mm |
| Levofloxacin | Disk diffusion | 34 mm | S | S ≥17 mm; I 14–16 mm; R ≤13 mm |

**Valvular necrotic tissue isolate**

| **Antimicrobial agent** | **Method** | **Recorded result** | **Recorded interpretation** | **CLSI M100 (32nd ed.) breakpoint range** |
| --- | --- | --- | --- | --- |
| Vancomycin | Disk diffusion | 24 mm | S | S ≥17 mm |
| Linezolid | Disk diffusion | 40 mm | S | S ≥21 mm |
| Chloramphenicol | Disk diffusion | 32 mm | S | S ≥21 mm; I 18–20 mm; R ≤17 mm |
| Penicillin G | Broth microdilution | 0.032 μg/mL | S | S ≤0.12 μg/mL; I 0.25–2 μg/mL; R ≥4 μg/mL |
| Clindamycin | Disk diffusion | 34 mm | S | S ≥19 mm; I 16–18 mm; R ≤15 mm |
| Ceftriaxone | Disk diffusion | 34 mm | S | S ≥27 mm; I 25–26 mm; R ≤24 mm |
| Erythromycin | Disk diffusion | 40 mm | S | S ≥21 mm; I 16–20 mm; R ≤15 mm |
| Levofloxacin | Disk diffusion | 34 mm | S | S ≥17 mm; I 14–16 mm; R ≤13 mm |

S, susceptible; MIC, minimum inhibitory concentration. Susceptibility categories are reproduced from the archived clinical laboratory report, which applied CLSI M100, 32nd edition. The report retained disk-diffusion zone diameters for seven agents and broth-microdilution MICs only for penicillin G; additional MIC values were not available for retrospective analysis.
