# Supplementary material for: Comparative genomics identifies a cyl-like region in Streptococcus sinensis isolates from a patient with infective endocarditis
Source: Front Microbiol. 2026 Oct 19;17:1990134. doi: 10.3389/fmicb.2026.1990134 (PMC13724373; doi:10.3389/fmicb.2026.1990134)
Supplement: Supplementary file 4 [file Table_2.docx]

**Supplementary Table S5. Targeted BLAST, read-mapping, and Pfam/HMMER verification of the *cyl*-like block**

Scope. The reference queries were the 12 coding sequences of the public *S. sinensis* SSSC01 locus (GCF_030586725.1). BLAST alignment metrics were identical in 418A, 418B, and 433 and are therefore consolidated in Table S5A. Table S5B reports isolate-specific read-mapping support. Because every clinical coding sequence was full-length and nucleotide-identical to SSSC01, the corresponding public SSSC01 proteins were used for the Pfam scan in Table S5C. These analyses verify sequence conservation, read support, and broad domain architecture only; they do not establish expression, enzymatic activity, hemolysis, or virulence.

**Table S5A. Gene-specific BLAST verification**

BLAST+ v2.15.0; E-value threshold 1 × 10−5; other parameters default. All 12 coding sequences in each clinical isolate had 100% query coverage, 100% subject coverage, and 100% nucleotide identity relative to SSSC01. The complete block spans Scaffold10 20,397–31,737 (11,341 bp).

| **Gene** | **Clinical isolates** | **Scaffold** | **Coordinates** | **Strand** | **Alignment (bp)** | **Identity (%)** | **Query cov. (%)** | **Subject cov. (%)** | **E-value** | **Bit score** |
| --- | --- | --- | --- | --- | --- | --- | --- | --- | --- | --- |
| *cylX* | 418A/418B/433 | Scaffold10 | 20397–20705 | + | 309 | 100 | 100 | 100 | 3.32E-163 | 571 |
| *cylD* | 418A/418B/433 | Scaffold10 | 20702–21550 | + | 849 | 100 | 100 | 100 | 0 | 1568 |
| *cylG* | 418A/418B/433 | Scaffold10 | 21547–22269 | + | 723 | 100 | 100 | 100 | 0 | 1336 |
| *acpC* | 418A/418B/433 | Scaffold10 | 22262–22564 | + | 303 | 100 | 100 | 100 | 7.03E-160 | 560 |
| *cylZ* | 418A/418B/433 | Scaffold10 | 22554–23027 | + | 474 | 100 | 100 | 100 | 0 | 876 |
| *cylA* | 418A/418B/433 | Scaffold10 | 23017–23946 | + | 930 | 100 | 100 | 100 | 0 | 1718 |
| *cylB* | 418A/418B/433 | Scaffold10 | 23939–24817 | + | 879 | 100 | 100 | 100 | 0 | 1624 |
| *cylE* | 418A/418B/433 | Scaffold10 | 24814–26823 | + | 2010 | 100 | 100 | 100 | 0 | 3712 |
| *cylF* | 418A/418B/433 | Scaffold10 | 26807–27760 | + | 954 | 100 | 100 | 100 | 0 | 1762 |
| *cylI* | 418A/418B/433 | Scaffold10 | 27757–29949 | + | 2193 | 100 | 100 | 100 | 0 | 4050 |
| *cylJ* | 418A/418B/433 | Scaffold10 | 29958–31181 | + | 1224 | 100 | 100 | 100 | 0 | 2261 |
| *cylK* | 418A/418B/433 | Scaffold10 | 31132–31737 | + | 606 | 100 | 100 | 100 | 0 | 1120 |

**Table S5B. Isolate-specific read-mapping support**

Quality-controlled reads were aligned to the corresponding assembly with BWA-MEM v0.7.17 using default parameters; depth was calculated with SAMtools (version not supplied). Scaffold10 was 84,521 bp in all three assemblies. The complete block lies 20,396 bp from the left scaffold end and 52,784 bp from the right scaffold end. Coverage of 100% denotes a coverage fraction of 1.0 in the company-supplied table.

| **Gene** | **Isolate** | **Scaffold** | **Scaffold length (bp)** | **Coordinates** | **Mean depth (×)** | **Coverage (%)** | **Interpretation** |
| --- | --- | --- | --- | --- | --- | --- | --- |
| *cylX* | 418A | Scaffold10 | 84521 | 20397–20705 | 659.6 | 100 | Fully covered |
| *cylX* | 418B | Scaffold10 | 84521 | 20397–20705 | 702.4 | 100 | Fully covered |
| *cylX* | 433 | Scaffold10 | 84521 | 20397–20705 | 227.4 | 100 | Fully covered |
| *cylD* | 418A | Scaffold10 | 84521 | 20702–21550 | 665.8 | 100 | Fully covered |
| *cylD* | 418B | Scaffold10 | 84521 | 20702–21550 | 692.2 | 100 | Fully covered |
| *cylD* | 433 | Scaffold10 | 84521 | 20702–21550 | 229.0 | 100 | Fully covered |
| *cylG* | 418A | Scaffold10 | 84521 | 21547–22269 | 645.6 | 100 | Fully covered |
| *cylG* | 418B | Scaffold10 | 84521 | 21547–22269 | 613.4 | 100 | Fully covered |
| *cylG* | 433 | Scaffold10 | 84521 | 21547–22269 | 250.6 | 100 | Fully covered |
| *acpC* | 418A | Scaffold10 | 84521 | 22262–22564 | 638.6 | 100 | Fully covered |
| *acpC* | 418B | Scaffold10 | 84521 | 22262–22564 | 623.5 | 100 | Fully covered |
| *acpC* | 433 | Scaffold10 | 84521 | 22262–22564 | 221.9 | 100 | Fully covered |
| *cylZ* | 418A | Scaffold10 | 84521 | 22554–23027 | 571.6 | 100 | Fully covered |
| *cylZ* | 418B | Scaffold10 | 84521 | 22554–23027 | 549.0 | 100 | Fully covered |
| *cylZ* | 433 | Scaffold10 | 84521 | 22554–23027 | 226.8 | 100 | Fully covered |
| *cylA* | 418A | Scaffold10 | 84521 | 23017–23946 | 678.4 | 100 | Fully covered |
| *cylA* | 418B | Scaffold10 | 84521 | 23017–23946 | 641.0 | 100 | Fully covered |
| *cylA* | 433 | Scaffold10 | 84521 | 23017–23946 | 249.6 | 100 | Fully covered |
| *cylB* | 418A | Scaffold10 | 84521 | 23939–24817 | 635.4 | 100 | Fully covered |
| *cylB* | 418B | Scaffold10 | 84521 | 23939–24817 | 672.2 | 100 | Fully covered |
| *cylB* | 433 | Scaffold10 | 84521 | 23939–24817 | 218.6 | 100 | Fully covered |
| *cylE* | 418A | Scaffold10 | 84521 | 24814–26823 | 624.5 | 100 | Fully covered |
| *cylE* | 418B | Scaffold10 | 84521 | 24814–26823 | 661.1 | 100 | Fully covered |
| *cylE* | 433 | Scaffold10 | 84521 | 24814–26823 | 222.4 | 100 | Fully covered |
| *cylF* | 418A | Scaffold10 | 84521 | 26807–27760 | 568.6 | 100 | Fully covered |
| *cylF* | 418B | Scaffold10 | 84521 | 26807–27760 | 615.9 | 100 | Fully covered |
| *cylF* | 433 | Scaffold10 | 84521 | 26807–27760 | 211.1 | 100 | Fully covered |
| *cylI* | 418A | Scaffold10 | 84521 | 27757–29949 | 632.4 | 100 | Fully covered |
| *cylI* | 418B | Scaffold10 | 84521 | 27757–29949 | 628.7 | 100 | Fully covered |
| *cylI* | 433 | Scaffold10 | 84521 | 27757–29949 | 239.2 | 100 | Fully covered |
| *cylJ* | 418A | Scaffold10 | 84521 | 29958–31181 | 664.9 | 100 | Fully covered |
| *cylJ* | 418B | Scaffold10 | 84521 | 29958–31181 | 668.3 | 100 | Fully covered |
| *cylJ* | 433 | Scaffold10 | 84521 | 29958–31181 | 234.6 | 100 | Fully covered |
| *cylK* | 418A | Scaffold10 | 84521 | 31132–31737 | 579.4 | 100 | Fully covered |
| *cylK* | 418B | Scaffold10 | 84521 | 31132–31737 | 567.3 | 100 | Fully covered |
| *cylK* | 433 | Scaffold10 | 84521 | 31132–31737 | 210.3 | 100 | Fully covered |

**Table S5C. Pfam profile-HMM results**

EMBL-EBI HMMER web API hmmscan against Pfam 37.2 (24,076 profile models), accessed 20 September 2026. A match was accepted only if both the full-sequence score and the best-domain score met the model-specific Pfam gathering thresholds (GA). Query coverage is aligned query length divided by protein length; model coverage is aligned HMM length divided by profile length. Multiple passing profiles may overlap. Pfam did not provide a dedicated gene-specific model for every *cyl* component; no qualifying profile does not indicate gene absence.

| **Gene** | **Protein ID** | **aa** | **Pfam accession** | **Profile** | **Full-seq E-value** | **Full score** | **Domain i-Evalue** | **Domain score** | **Query cov. (%)** | **Model cov. (%)** | **GA seq/dom** | **Decision** |
| --- | --- | --- | --- | --- | --- | --- | --- | --- | --- | --- | --- | --- |
| *cylX* | WP_304949056.1 | 102 | PF20441.4 | TerL_nuclease | 0.13 | 11.81 | 0.13 | 11.76 | 60.78 | 20.63 | 24.00/24.00 | Best reported; below ≥1 GA threshold |
| *cylD* | WP_304949057.1 | 282 | PF20126.4 | TumE | 0.04 | 14.46 | 0.08 | 13.44 | 13.12 | 41.98 | 23.40/23.40 | Best reported; below ≥1 GA threshold |
| *cylG* | WP_304949058.1 | 240 | PF13561.12 | adh_short_C2 | 2.59e-64 | 217.44 | 3.03e-64 | 217.21 | 94.58 | 97.46 | 27.00/27.00 | Pass both GA thresholds |
| *cylG* | WP_304949058.1 | 240 | PF00106.31 | adh_short | 2.40e-57 | 193.98 | 2.82e-57 | 193.75 | 80.00 | 99.49 | 26.60/25.00 | Pass both GA thresholds |
| *cylG* | WP_304949058.1 | 240 | PF08659.16 | KR | 2.36e-22 | 80.14 | 3.44e-22 | 79.61 | 62.50 | 84.44 | 22.60/22.60 | Pass both GA thresholds |
| *cylG* | WP_304949058.1 | 240 | PF01370.27 | Epimerase | 2.59e-09 | 37.26 | 5.42e-09 | 36.21 | 58.75 | 51.25 | 20.90/20.90 | Pass both GA thresholds |
| *acpC* | WP_304949059.1 | 100 | PF00550.31 | PP-binding | 1.28e-07 | 32.17 | 1.68e-07 | 31.80 | 70.00 | 98.51 | 20.80/20.80 | Pass both GA thresholds |
| *cylZ* | WP_304949060.1 | 157 | PF07977.19 | FabA | 4.52e-23 | 81.80 | 9.90e-23 | 80.69 | 68.79 | 76.81 | 23.00/23.00 | Pass both GA thresholds |
| *cylA* | WP_304949061.1 | 309 | PF00005.33 | ABC_tran | 3.04e-34 | 118.94 | 7.78e-33 | 114.38 | 46.93 | 100.00 | 25.00/25.00 | Pass both GA thresholds |
| *cylB* | WP_304949062.1 | 292 | PF12698.13 | ABC2_membrane_3 | 3.18e-10 | 40.05 | 3.18e-10 | 40.05 | 77.40 | 53.60 | 31.60/31.60 | Pass both GA thresholds |
| *cylB* | WP_304949062.1 | 292 | PF01061.29 | ABC2_membrane | 5.67e-09 | 36.22 | 5.67e-09 | 36.22 | 74.66 | 93.84 | 26.90/26.90 | Pass both GA thresholds |
| *cylE* | WP_304949063.1 | 669 | PF13420.13 | Acetyltransf_4 | 3.88e-06 | 27.38 | 1.05e-04 | 22.73 | 19.88 | 87.10 | 24.30/24.30 | Best reported; below ≥1 GA threshold |
| *cylF* | WP_304949064.1 | 317 | PF01571.26 | GCV_T | 2.97e-20 | 72.97 | 4.77e-20 | 72.29 | 59.94 | 79.61 | 25.60/25.60 | Pass both GA thresholds |
| *cylI* | WP_304949065.1 | 730 | PF00109.32 | ketoacyl-synt | 1.41e-36 | 126.79 | 2.41e-24 | 86.71 | 30.82 | 97.62 | 20.80/20.80 | Pass both GA thresholds |
| *cylI* | WP_304949065.1 | 730 | PF02801.28 | Ketoacyl-synt_C | 8.64e-31 | 106.81 | 3.57e-30 | 104.82 | 15.34 | 99.16 | 20.80/20.80 | Pass both GA thresholds |
| *cylJ* | WP_304949066.1 | 407 | PF00201.24 | UDPGT | 2.53e-06 | 26.98 | 4.64e-06 | 26.11 | 19.16 | 15.83 | 19.50/19.50 | Pass both GA thresholds |
| *cylJ* | WP_304949066.1 | 407 | PF04101.22 | Glyco_tran_28_C | 1.34e-05 | 25.66 | 4.64e-05 | 23.90 | 21.38 | 53.01 | 22.00/22.00 | Pass both GA thresholds |
| *cylK* | WP_304949067.1 | 201 | n.a. | n.a. | n.a. | n.a. | n.a. | n.a. | n.a. | n.a. | n.a. | No Pfam profile reported |
