# Supplementary material for: Comparative genomics identifies a cyl-like region in Streptococcus sinensis isolates from a patient with infective endocarditis
Source: Front Microbiol. 2026 Oct 19;17:1990134. doi: 10.3389/fmicb.2026.1990134 (PMC13724373; doi:10.3389/fmicb.2026.1990134)
Supplement: Supplementary file 5 [file Table_3.docx]

**Supplementary Table S6. Raw OD_541_ readings and exploratory PBS-corrected, SDS-normalized signals.**

| **Condition** | **Tube 1** | **Tube 2** | **Tube 3** | **Mean ± SD** | **Relative signal (%)** |
| --- | --- | --- | --- | --- | --- |
| PBS + sheep erythrocytes | 0.11 | 0.08 | 0.06 | 0.083 ± 0.025 | 0.0 (negative reference) |
| 0.1% SDS before 1:1 mixing | 1.37 | 1.27 | 1.16 | 1.267 ± 0.105 | 100.0 (positive reference) |
| *S. agalactiae* control | 0.64 | 0.51 | 0.35 | 0.500 ± 0.145 | 35.2 |
| 418A | 0.39 | 0.42 | 0.38 | 0.397 ± 0.021 | 26.5 |
| 418B | 0.87 | 0.54 | 0.43 | 0.613 ± 0.229 | 44.8 |
| 433 | 0.47 | 0.20 | 0.31 | 0.327 ± 0.136 | 20.6 |

Values are three technical tubes from one experiment. The mean relative signal was calculated as 100 × (mean sample OD_541_ − mean PBS OD_541_)/(mean SDS-control OD_541_ − mean PBS OD_541_). The SDS solution was 0.1% before 1:1 mixing (0.05% final, assuming equal-volume mixing). These values are relative supernatant OD_541_ signals, not validated percentages of erythrocyte lysis. No bacteria-only optical blank was recorded, and the control strain accession/identifier was not recorded.
